# Buccal injection of synthetic HPV long peptide vaccine induces local and systemic antigen-specific CD8+ T-cell immune responses and antitumor effects without adjuvant

**DOI:** 10.1186/s13578-016-0083-9

**Published:** 2016-03-03

**Authors:** Ming-Chieh Yang, Andrew Yang, Jin Qiu, Benjamin Yang, Liangmei He, Ya-Chea Tsai, Jessica Jeang, T.-C. Wu, Chien-Fu Hung

**Affiliations:** 1 Department of Pathology, Johns Hopkins Medical Institutions, Baltimore, MD USA; 2 Department of Surgery, Kaohsiung Veterans General Hospital, Kaohsiung, Taiwan China; 3 Department of Obstetrics and Gynecology, Shanghai Tenth People’s Hospital of Tongji University, Shanghai, China; 4 Department of Obstetrics and Gynecology, Johns Hopkins Medical Institutions, Baltimore, MD USA; 5 Department of Molecular Microbiology and Immunology, Johns Hopkins Medical Institutions, Baltimore, MD USA; 6 Department of Oncology, Johns Hopkins Medical Institutions, Baltimore, MD USA; 7 Departments of Pathology and Oncology, The Johns Hopkins University School of Medicine, CRB II Room 307, 1550 Orleans Street, Baltimore, MD 21231 USA

**Keywords:** Immunotherapy, E7 long peptide, Adjuvant free, Buccal tumor

## Abstract

**Background:**

Human Papillomavirus is responsible for over 99 % of cervical cancers and is associated with cancers of the head and neck. The currently available prophylactic vaccines against HPV do not generate therapeutic effects against established HPV infections and associated lesions and disease. Thus, the need for a therapeutic vaccine capable of treating HPV-induced malignancies persists. Synthetic long peptides vaccination is a popular antigen delivery method because of its safety, stability, production feasibility, and its need to be processed by professional antigen presenting cells before it can be presented to cytotoxic CD8+ T lymphocytes. Cancers in the buccal mucosa have been shown to elicit cancer-related inflammations that are capable of recruiting inflammatory and immune cells to generate antitumor effects. In the current study, we evaluated the therapeutic potential of synthetic HPV long peptide vaccination in the absence of adjuvant in the TC-1 buccal tumor model.

**Result:**

We show that intratumoral vaccination with E7 long peptide alone effectively controls buccal TC-1 tumors in mice. Furthermore, we observed an increase in systemic as well as local E7-specific CD8+ T cells in buccal tumor-bearing mice following the vaccination. Finally, we show that induction of immune responses against buccal tumors by intratumoral E7 long peptide vaccination is independent of CD4+ T cells, and that the phenomenon may be related to the unique environment associated with mucosal tissues.

**Conclusion:**

Our results suggest the possibility for clinical translation of the administration of adjuvant free therapeutic long peptide vaccine as a potentially effective and safe strategy for mucosal HPV-associated tumor treatment.

**Electronic supplementary material:**

The online version of this article (doi:10.1186/s13578-016-0083-9) contains supplementary material, which is available to authorized users.

## Background

It is now clear that human papillomavirus (HPV) infection is responsible for over 99 % of all cervical cancers, and is also associated with many other anogenital malignancies including vaginal and anal cancers [1]. In addition, the prevalence of HPV infection in head and neck cancers increased significantly within the past decade, with approximately 75 % of diagnosed oropharyngeal cancers corresponding with HPV infection [2]. Among all HPV subtypes, the high-risk oncogenic HPV subtypes, predominantly HPV type 16, are responsible for the majority of HPV associated cancer [3, 4]. The known etiology of HPV-associated diseases provides an excellent opportunity to develop vaccines against the high-risk HPV types. Encouragingly, there have been several successes in the development of prophylactic vaccines against disease-causing HPV subtypes [5]. However, these prophylactic vaccines can only prevent infections and do not generate therapeutic effects against established HPV infections and HPV-associated lesions [6]. Thus, the urgent need for the development of a therapeutic vaccine capable of treating HPV-induced malignancies persists.

To date, several clinical trials have been conducted using HPV-16 encoded oncoproteins E6 and E7 as targets of immunotherapy to treat HPV-induced cancers [7–10]. Among different therapeutic vaccine designs, peptide-based vaccines containing minimal epitopes of oncoproteins E6 and E7 have been popular and promising due to their safety, stability, and production feasibility [9, 11–13]. However, some limitations to peptide vaccines dampen their application efficacy. Importantly, short peptides may be directly loaded onto any MHC I molecules on the surface of cells, including those that are not professional antigen presenting cells (APCs). This may result in interaction between T-cell receptor and MHC I—antigen peptide complex in the absence of co-stimulation, causing T-cell anergy and immune tolerance [13]. To overcome this issue and increase the efficacy of the peptide vaccine, the length of the peptide antigen has been increased [14, 15]. The synthetic long peptides are too large for the direct loading onto MHC I molecules on the surface of cells, thus requiring the peptide to be taken up and processed before the epitope can be presented on MHC I molecules, which is a process unique to the professional APCs. The professional APCs, such as the dendritic cells (DCs), can also provide the co-stimulatory signals during antigen presentation, ensuring quality T cell activation [16, 17].

Despite the improved antigen presentation process of the synthetic long peptide vaccine, the issue of poor immunogenicity remains. Typically, additional adjuvant or immunostimulant is required to induce the desired immune responses for vaccines incorporating synthetic peptides of a target antigen [9]. It is well known that malignant tumors, including squamous cell carcinomas of head and neck, are strongly associated with local inflammation [18, 19]. These cancer-related inflammations trigger the release of cytokines and the recruitment of inflammatory and immune cells, which could lead to the induction of either immune suppression or anti-tumor immunity [20, 21]. Thus, the inflammatory nature of cancer may potentially serves as self-adjuvant capable of inducing the antigen-specific immune responses following synthetic long peptide vaccination.

In the current study, we evaluated the therapeutic potential of a synthetic HPV long peptide vaccine in the absence of adjuvant in the TC-1 buccal tumor model. We showed that intratumoral (I.T.) vaccination with HPV-16 E7aa 43-62 synthetic long peptide lead to enhanced antitumor effect in buccal tumor-bearing mice in the absence of adjuvant administration. Furthermore, we observed an increase in the number of E7-specific CD8+ T cells in the peripheral blood, spleen, and the buccal mucosa tissue. We also observed that the antitumor effect of the synthetic long peptide vaccination is CD8+ T cells dependent and CD4+ T cells independent. We also showed that in comparison to subcutaneous tumor model, intratumoral synthetic long peptide vaccination in the absence of adjuvant lead to the generation of superior E7-specific CD8+ T cell response as well as more potent therapeutic antitumor effects against tumors located in the buccal mucosal region. Finally, we demonstrated that the observed therapeutic effects generated by intratumoral E7 long peptide vaccination in the buccal area are abolished upon deletion of toll-like receptor 4. Our results indicate that adjuvant free therapeutic long peptide vaccination is an effective and safe therapeutic strategy for treating tumors located in the mucosa.

## Results

### Intratumoral administration of synthetic HPV long peptide vaccine in the buccal area generates potent antitumor responses

**Fig. 1:**
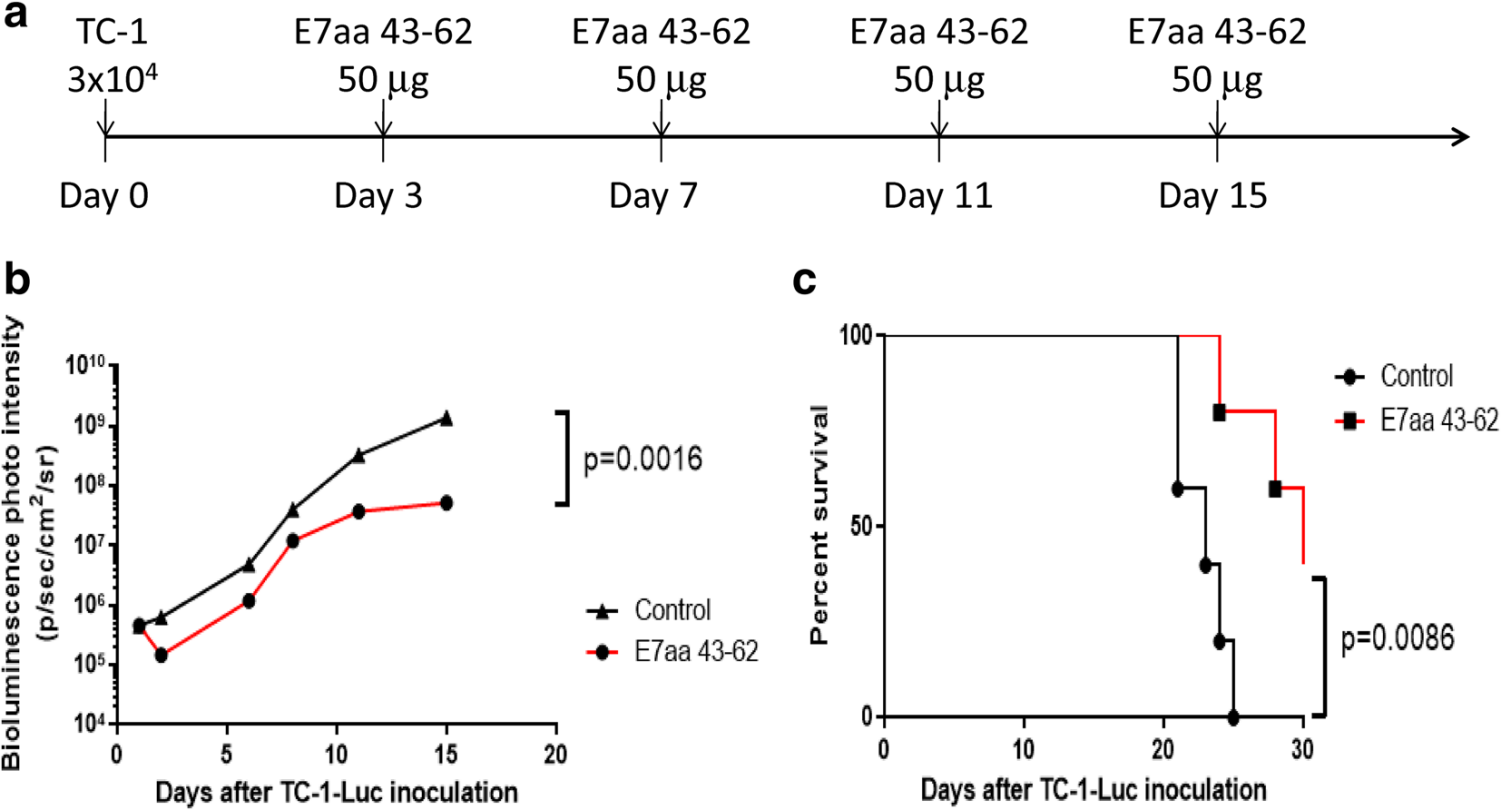
Characterization of antitumor effect in tumor bearing mice treated with intratumoral synthetic HPV-16 E7aa 43-62 long peptide vaccination in buccal mucosal region. 3 × 10^4^ TC-1-Luc cells were submucosally injected into the right buccal area of C57BL/6 mice (five per group). Three days after tumor injection, mice were vaccinated intratumorally with or without 50 μg of synthetic HPV-16 E7aa 43-62 peptide for four times in a 4-day intervals. **a** Schematic diagram of treatment regimens. **b** Line graph depicting the change in mean luminescence intensity of tumor bearing mice after tumor injection (mean ± SD). **c** Kaplan–Meier survival analysis of mice

First, we assessed the antitumor effect generated by intratumoral synthetic HPV-16 E7aa 43-62 peptide vaccination in the HPV-16 E7-expressing TC-1 buccal tumor model. C57BL/6 mice (five per group) were challenged with 3 × 10^4^ TC-1-Luc cells in the right buccal area, then vaccinated I.T. with or without synthetic HPV-16 E7aa 43-62 peptide 3 days after for 4 times with a 4 day interval (Fig. 1a). As shown in Fig. 1b, mice vaccinated with E7 peptide generated significantly better antitumor effects which resulted in tumor control as measured by bioluminescence intensity compared to untreated mice. In addition, the vaccinated mice demonstrated longer survival compared to untreated mice (Fig. 1c). However, when the tumor bearing mice were vaccinated with non-specific CTL peptide (OVA 241-270), neither effective tumor control (Additional file 1: Figure S1A–B) nor prolonged survival (Additional file 1: Figure S1C) were observed. These data indicated that intratumoral synthetic E7 long peptide vaccination leads to effective E7-expressing buccal tumor control in the absence of additional adjuvant.

### Intratumoral administration of synthetic HPV long peptide vaccine in the buccal area leads to generation of systemic and local E7-specific CD8+ T cell responses

**Fig. 2:**
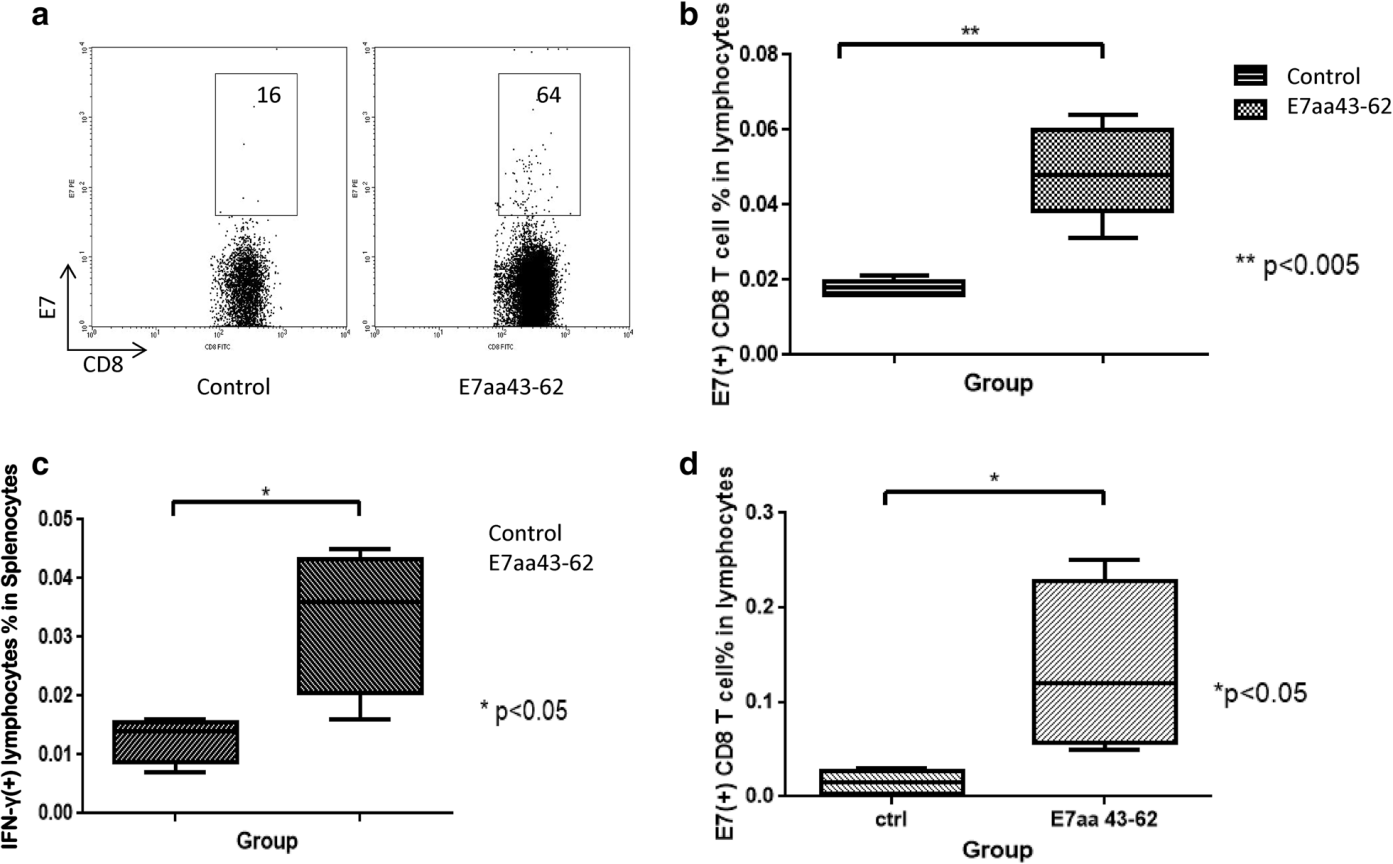
Characterization of systemic E7-specific CD8+ T cells and local E7-specific activated CD8+ T cells in tumor bearing mice. 3 × 10^4^ TC-1-Luc cells were submucosally injected into the right buccal area of C57BL/6 mice (five per group). Three days after tumor injection, mice were vaccinated intratumorally with or without 50 μg of synthetic HPV-16 E7aa 43-62 peptide for four times in a 4-day intervals. 21 days after tumor injection, peripheral blood was collected and the spleen and tumor were harvested. Cells obtained from the peripheral blood and tumor were stained with PE-conjugated HPV16 H-2D-RAHYNIVTF tetramer and APC-conjugated CD8 monoclonal antibody followed by flow cytometry analysis. Spleenocytes were stimulated with HPV16 E7aa49-57 peptide in the presence of GolgiPlug and IFN-γ-secreting CD8+ T cells were detected by intracellular cytokine staining followed by a flow cytometry analysis. **a** Representative flow cytometry images showing the amount of E7-specific CD8+ T cells per 1 × 10^5^ lymphocytes in the peripheral blood of various groups. **b** Bar graph depicting the amount of E7-specific CD8+ T cells per 1 × 10^5^ lymphocytes in the peripheral blood of various groups (mean ± SD). **c** Bar graph depicting the amount of IFN-γ positive lymphocytes per 1 × 10^5^ splenocytes (mean ± SD). **d** Bar graph depicting the percentage of E7-specific CD8+ T cells in all lymphocytes in the buccal tumor (mean ± SD)

We next evaluate the potential of I.T. E7aa 43-62 long peptide vaccination in generating antigen-specific adaptive immune responses. C57BL/6 mice (five per group) were challenged with 3 × 10^4^ TC-1-Luc cells in the right buccal area, then vaccinated I.T. with or without synthetic E7aa 43-62 peptide 3 days after for four times with a 4 day interval. Peripheral blood, spleen, and tumors of the tumor bearing mice were harvested 21 days after tumor challenge and either stained with E7 tetramer or stimulated with E7 peptide followed by IFN-γ staining and analyzed by flow cytometry. As shown in Fig. 2a, b, more E7-specific CD8+ T cells were induced in the peripheral blood of tumor bearing mice vaccinated with E7aa 43-62 long peptide compare to mice without vaccination. Mice vaccinated with E7aa 43-62 long peptide also generated more IFN-γ secreting CD8+ T cells in spleen compared to untreated mice (Fig. 2c). Furthermore, as shown in Fig. 2d, mice vaccinated with E7aa 43-62 long peptide also generated more E7-specific CD8+ in the buccal tumor compared to untreated mice. Taken together, these data suggest that intratumoral synthetic long peptide vaccination can induce potent systemic E7-specific CD8+ T cell generation as well as local antigen-specific CD8+ T cells accumulation in buccal tumor in the absence of adjuvant.

### The synthetic HPV long peptide vaccine generates potent antitumor effects against HPV-16 E7 expressing tumors in CD4-depleted mice but not in CD8-depleted mice

**Fig. 3:**
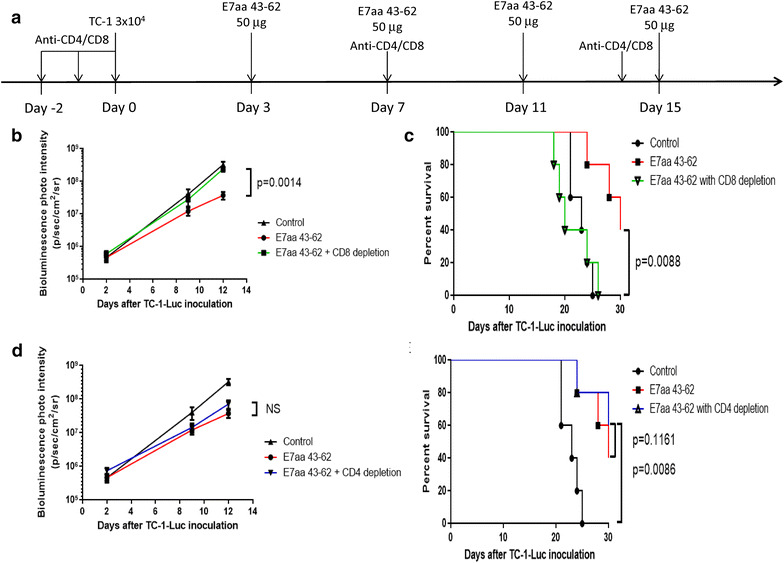
Effect of CD4+ or CD8+ T cell depletion on the antitumor response of synthetic HPV-16 E7aa 43-62 long peptide vaccine. 100 μg of purified rat monoclonal antibody GK1.5 (anti-CD4) or mAB 2.43 (anti-CD8) were injected intraperitoneally 2 days before tumor injection. The injections were repeated once per day for 2 days until the day of tumor challenge. On the day of tumor challenge, 3 × 10^4^ TC-1-Luc cells were submucosally injected into the right buccal area of C57BL/6 mice (five per group). Three days after tumor injection, mice were vaccinated intratumorally with or without 50 μg of synthetic HPV-16 E7aa 43-62 peptide for four times in a 4-day intervals. Mice also continued to receive anti-CD4 or anti-CD8 antibody injections once every week after tumor injection. **a** Schematic diagram of treatment regimens. **b** Line graph depicting the change in mean luminescence intensity of tumor bearing mice after tumor injection with or without CD8 depletion (mean ± SD). **c** Kaplan–Meier survival analysis of mice in CD8 depletion experiment. **d** Line graph depicting the change in mean luminescence intensity of tumor bearing mice after tumor injection with or without CD4 depletion (mean ± SD). **e** Kaplan–Meier survival analysis of mice in CD4 depletion experiment. *NS* indicates not significant

Next, we sought to determine which immune cell population is predominately responsible for the generation of antitumor effects following synthetic E7 long peptide vaccination. We repeated the experiment in both CD4 depleted mice and CD8 depleted mice. As shown in Fig. 3b, c, CD8 depleted mice showed significantly increased tumor growth as measured by bioluminescence intensity as well as reduction in survival even after synthetic long peptide vaccination compared to CD8 intact mice, at a level similar to the untreated mice. However, the treatment effects of tumor reduction and prolonged survival were observed in CD4 depleted, E7 long peptide vaccinated mice compared to mice without CD4 depletion (Fig. 3d, e). These results indicate that the antitumor responses elicited by I.T. vaccination of E7 long peptide in the absence of adjuvants are CD8+ T cells dependent and CD4+ T cells independent.

### Intratumoral administration of synthetic HPV long peptide vaccine leads to better generation of E7-specific CD8+ T cells and more potent antitumor effects against buccal mucosal tumor compared to subcutaneous tumor

**Fig. 4:**
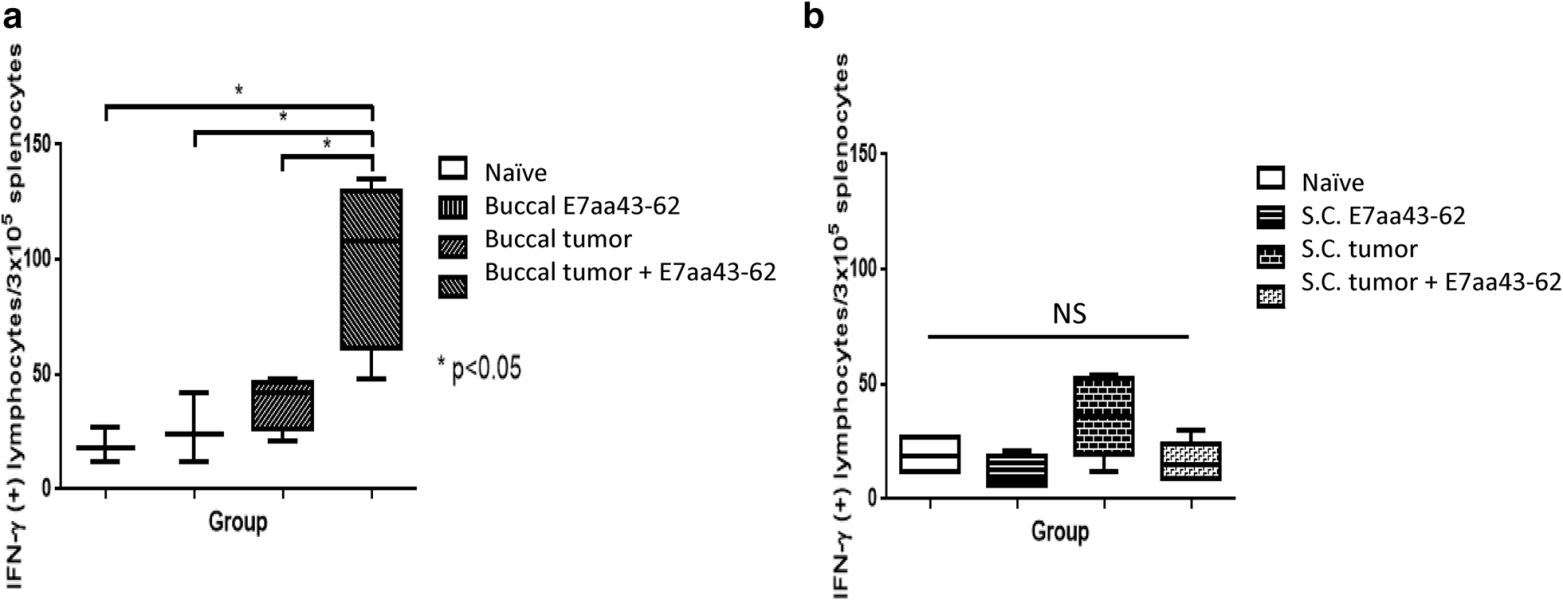
Comparison of HPV-16 E7 specific CD8+ T cell responses induced by synthetic HPV-16 E7aa 43-62 long peptide vaccine in various tumor model. C57BL/6 mice (five per group) received either 3 × 10^4^ TC-1-Luc cells injection submucosally into the right buccal area or 1 × 10^5^ TC-1-Luc cells injection subcutaneously into the abdomen. Three days after tumor injection, mice were vaccinated intratumorally with or without 50 μg of synthetic HPV-16 E7aa 43-62 peptide for four times in a 4-day intervals. 21 days after tumor injection, spleenocytes were harvested and stimulated with HPV16 E7aa49-57 peptide in the presence of GolgiPlug and IFN-γ-secreting CD8+ T cells were detected by intracellular cytokine staining followed by a flow cytometry analysis. **a** Bar graph depicting the amount of IFN-γ positive lymphocytes per 3 × 10^5^ splenocytes for buccal treatments (mean ± SD). **b** Bar graph depicting the amount of IFN-γ positive lymphocytes per 3 × 10^5^ splenocytes for subcutaneous treatments (mean ± SD). *NS* indicates not significant

**Fig. 5:**
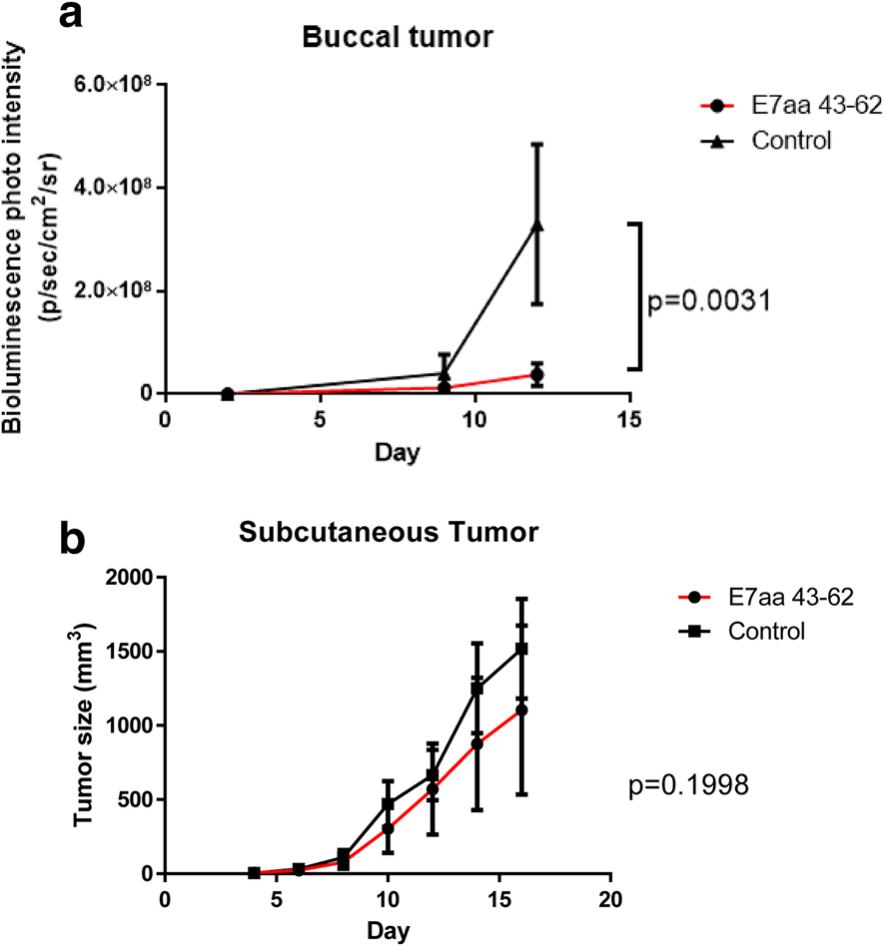
Comparison of antitumor effects induced by synthetic HPV-16 E7aa 43-62 long peptide vaccine in various tumor model. C57BL/6 mice (five per group) received either 3 × 10^4^ TC-1-Luc cells injection submucosally into the right buccal area or 1 × 10^5^ TC-1-Luc cells injection subcutaneously into the abdomen. Three days after tumor injection, mice were vaccinated intratumorally with or without 50 μg of synthetic HPV-16 E7aa 43-62 peptide for four times in a 4-day intervals. **a** Line graph depicting the change in mean luminescence intensity of tumor bearing mice after buccal tumor injection (mean ± SD). **b** Line graph depicting the change in mean luminescence intensity of tumor bearing mice after subcutaneous tumor injection (mean ± SD)

Finally, we tested whether I.T. synthetic E7 long peptide vaccination without adjuvant can also elicit an antitumor immune response in other tumor models. C57BL/6 mice (five per group) were challenged with TC-1-Luc cells either submucosally in the right buccal area or subcutaneously in the abdomen and then vaccinated I.T. with synthetic E7 long peptide. 21 days after tumor challenges, the spleen of mice were harvested and stimulated with E7 peptide followed by IFN-γ staining and analyzed by flow cytometry. As shown in Fig. 4a, b, I.T. synthetic long peptide vaccination in buccal tumor-bearing mice generated significantly higher amount of IFN-γ secreting CD8+ T cells in the spleen compared to naïve mice, mice received buccal vaccination alone or buccal tumor challenge alone, whereas there were no significant differences in the number of IFN-γ secreting CD8+ T cells in spleen of mice receiving subcutaneous treatments. Furthermore, mice challenged with buccal tumor injection and treated with I.T. synthetic E7 long peptide vaccination showed significantly reduced tumor growth compared to mice without treatment, whereas I.T. synthetic E7 long peptide vaccination was not able to control tumor growth in mice challenged with subcutaneous tumor injection (Fig. 5a, b). These data demonstrated that I.T. synthetic E7 long peptide vaccination without adjuvant supplementation is more effective in the treatment of tumors located in the buccal mucosa than in the treatment of tumors located in the subcutaneous abdomen.

### Knocking out toll-like receptor 4 abolishes the therapeutic effect of intratumoral synthetic HPV long peptide vaccination

**Fig. 6:**
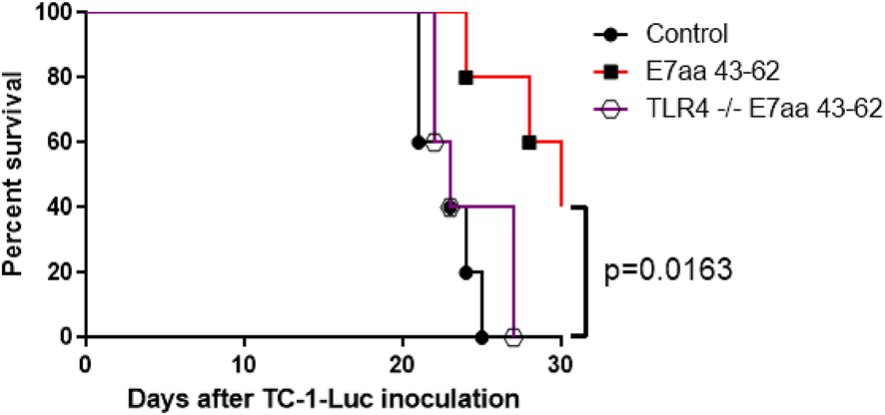
Effect of TLR4 Knockout on the antitumor response of synthetic HPV-16 E7aa 43-62 long peptide vaccine. 3 × 10^4^ TC-1-Luc cells were submucosally injected into the right buccal area of C57BL/6 or C57BL/10ScNJ (TLR4−/−) mice (five per group). Three days after tumor injection, mice were vaccinated intratumorally with or without 50 μg of synthetic HPV-16 E7aa 43-62 peptide for four times in a 4-day intervals. Figure represents the Kaplan–Meier survival analysis of mice in TLR4 knockout experiment

As mentioned in the introduction, the inflammatory nature of tumors located in the buccal area may serve as an adjuvant to induce antitumor immunity. Thus, the innate immune environment of the buccal area may play a role in mediating the generation of antigen-specific CD8+ T cell responses and anti-tumor effects observed from previous experiments involving intratumoral synthetic E7 long peptide vaccination against buccal TC-1 tumors. To examine the importance of innate immune responses in the observed treatment effect, we repeated the same experiment described in Fig. 1 in C57BL/10ScNJ (TLR4 deficient) mice. As shown in Fig. 6, TLR4 −/− mice treated with intratumoral synthetic E7 long peptide vaccination did not generate significant antitumor effect against buccal TC-1 tumor to prolong survival compared to the control, untreated mice. Buccal tumor-bearing mice with intact TLR4 function and received intratumoror synthetic E7 long peptide vaccination, however, survived significantly longer than TLR4 deficient mice who received the same treatment. This observation supports the hypothesis that the innate immune system of the buccal area is important for the immune responses and anti-tumor effects elicited by intratumoral E7 long peptide vaccination against tumors located in the buccal mucosa area.

## Discussion

In the current study, we examined the effects of adjuvant free, I.T. synthetic E7 long peptide vaccination on the generation of antigen-specific immune responses and antitumor effects. We observed that following I.T. synthetic E7 long peptide vaccination, the buccal tumor-bearing mice exhibited significant increase in systemic and local E7-specific CD8+ T cells, effectively controlling the tumor growth. In addition, we found that the antitumor effects generated by E7 long peptide vaccine is predominantly mediated by the CD8+ T cells and not by CD4+ T cells. Finally, we show that I.T. synthetic E7 long peptide vaccination without adjuvant elicited a better E7-specific CD8+ T cell response and more potent antitumor effects against tumors located in the buccal mucosa than to tumors located in the subcutaneous abdomen.

Of note, we demonstrated a difference in the ability of E7 long peptide vaccine in generating potent antigen-specific immune responses and antitumor effects when vaccinated I.T. against buccal tumors compared to when vaccinated I.T. against subcutaneous tumors (Figs. 4 and 5). This observation is supported by previous data. We have previously explored the employment of a sulfated polysaccharide compound from red algae, carrageenan, as adjuvant to generate antigen-specific immune responses and antitumor effects following subcutaneous E7 peptide vaccination [22]. The experiment demonstrated that subcutaneous E7 peptide vaccination alone without carrageenan administration did not lead to generation and activation of E7-specific CD8+ T cell immune response, which correspond to limited protective and therapeutic antitumor effects. In a separate study, we explored the adjuvant effect of chemotherapy in eliciting antigen-specific antitumor response and showed that I.T. vaccination of E7 peptide without cisplatin administration did not lead to effective control of subcutaneous tumors or the generation of potent E7-specific immune responses [23]. We explored the potential reasons for the phenomena observed in these previous studies, and showed that there was significantly less CD11c+ DCs accumulation in tumor as well as DCs migration to the lymph nodes when vaccinated I.T. with E7 peptide only without administration of adjuvant [22, 23]. Furthermore, DCs isolated from mice treated with E7 peptide vaccine alone express significantly less costimulatory molecules compared to those isolated from mice treated with both peptide vaccination and adjuvant administration, which translates into a lower ability to activate E7-specific CD8+ T cells. In contrast, a previous study has shown that buccal immunization with measles virus nucleoprotein (NP) alone is capable of eliciting a NP-specific CD8+ CTL response [24]. Furthermore, the study observed a rapid recruitment of DCs into the buccal mucosa after NP vaccination. In this study, we showed that the innate immune system regulated by TLR4 plays a significant role in eliciting the anti-tumor responses against buccal TC-1 tumor (Fig. 6). The differences in the tissue environment and the ability to recruit APCs to the local area may account for the difference in the generation of immune responses and antitumor effects between I.T. vaccination against buccal tumor versus I.T. vaccination against subcutaneous tumor. The trafficking of immune cells to the tumor location before and after I.T. E7 peptide vaccination in the buccal mucosa or subcutaneous abdomen should be further explored in future studies.

One key finding of the current study is that the experiments involving synthetic E7 long peptide vaccination were conducted without administration of supplementing adjuvants. Many studies have been performed to explore different approaches to elicit potent immune responses and antitumor effects through adjuvant-free vaccination [25–27]. Even though administration of adjuvants can elicit stronger immune responses, many substances with adjuvant effects have also been shown to have negative impact on tumor treatment [28, 29], to cause T cells dysfunction and retention [30, 31], to have neurotoxicity [32], or to induce autoimmunity [33]. Thus, identifying appropriate adjuvants that are both safe and effective when incorporated into vaccination strategies is a significant concern. Our study suggests the potential utilization of the natural immunogenic characteristics of mucosal tissue to elicit potent antigen-specific immune responses as well as therapeutic antitumor effects without administration of adjuvants, thus reducing the safety concerns for vaccination.

## Conclusion

In summary, we found that intratumoral therapeutic synthetic E7 long peptide vaccination resulted in both systemic and local increase of antigen-specific CD8+ T cells in mice bearing buccal tumors without the need to administer additional adjuvants to enhance the immunogenicity of the vaccine. This study suggests the possibility of clinical translation of administration of an adjuvant-free therapeutic HPV vaccine to generate potent cell-mediated immune responses and antitumor effects against HPV-associated lesions while preventing potential complications caused by adjuvants.

## Methods

### Mice

Six- to eight-week-old female C57BL/6 mice were purchased from the Charles Rivers Laboratories (Frederick, MD, USA). Female C57BL/10ScNJ mice carrying a spontaneous deletion of Tlr4 gene were obtained from The Jackson Laboratory (Bar Harbor, Maine, USA). All animal procedures were performed according to approved protocols at the Johns Hopkins Institutional Animal Care and Use Committee in accordance with recommendations for the proper use and care of laboratory animals.

### Cells

TC-1 cells expressing luciferase (TC-1-Luc) were generated and cultured using methods described previously. [34, 35]

### Synthetic long peptide vaccine

The synthetic long-peptide vaccine used in this study, E7aa 43-62, consists of synthetic peptide resembling 43-62 amino acid peptide chain of HPV-16 E7 antigen. This synthetic peptide construct contains an H-2D^b^-restricted E7 epitope (aa 49-57), and its immunogenicity has been demonstrated in our previous study [36]. The synthetic peptide was prepared at 95 % purity. No additional adjuvant was included in the vaccine.

For non-specific peptide vaccination experiment, CTL peptide OVA 241-270 (SMLVLLPDEVSGLEQLESIINFEKLTEWTS(OVA30)) were used. This peptide has been previously described [14].

### In vivo tumor treatment experiments

For the establishment and treatment of orophoryngeal tumor, 3 × 10^4^ TC-1-Luc cells were submucosally injected into the right buccal area of C57BL/6 or C57BL/10ScNJ mice. Tumor growth was confirmed by IVIS2000 bioluminescence imaging system. Three days after tumor injection, mice were vaccinated intratumorally with 50 μg of synthetic HPV-16 E7aa 43-62 peptide or 50 μg of CTL peptide OVA 241-270. Mice received same booster vaccination at 4-day intervals for a total of three boosters. The luminescence intensity of tumor was measured routinely with IVIS imaging machine.

For the establishment and treatment of abdominal tumor, 1 × 10^5^ TC-1-Luc cells were subcutaneously injected into the abdomen of C57BL/6 mice. Mice then received intratumoral vaccination using the same treatment schedule as described for the orophoryngeal tumor model. Tumor growth was monitored routinely by palpation and inspection.

### CD4/CD8 depletion

Hundred microgram of purified rat monoclonal antibody GK1.5 (anti-CD4) or mAB 2.43 (anti-CD8) were injected intraperitoneally 2 days before tumor injection. The injections were repeated once per day for 2 days until the day of tumor challenge. Mice were then inoculated with TC-1-Luc cells and vaccinated with synthetic HPV-16 E7aa 43-62 peptide following the same treatment schedule as described earlier for the oropharyngeal tumor model. Mice continued to receive anti-CD4 or anti-CD8 antibody injections once every week after tumor injection.

### Peripheral blood cell, splenocyte, and tumor infiltrating lymphocyte preparation

Twenty-one days after tumor injection, peripheral blood was obtained from the mice treated with various treatment regimen and the spleen and TC-1 tumors of the mice were harvested. For the preparation of splenocytes, the spleen was meshed through a 70 μm nylon filter mesh. The splenocytes and peripheral blood cells were treated with ACK lysis buffer to lyse the red blood cells, the cells were then washed and viable cells were identified using trypan blue dye exclusion. TC-1 tumors were surgically excised using sterile technique, placed in RPMI-1640 medium containing 100 U/ml penicillin and 100 μg/ml streptomycin and washed with PBS. The solid tumors were then minced into 1- to 2-mm pieces and immersed in serum-free RPMI-1640 medium containing 0.05 mg/ml collagenase I, 0.05 mg/ml collagenase IV, 0.025 mg/ml hyaluronidase IV, 0.25 mg/ml DNase I, 100 U/ml penicillin, and 100 μg/ml streptomycin and incubated at 37 °C with periodic agitation. The tumor digest was then filtered through a 70 μm nylon filter mesh to remove undigested tissue fragments. The resultant single tumor cell suspensions and tumor-infiltrating lymphocytes were washed twice in Hank’s buffered salt solution (HBSS) (400 g for 10 min), and viable cells were determined using trypan blue dye exclusion.

### Tetramer analysis of E7-specific CD8+ T cells in tumor bearing mice

Cells harvested from the peripheral blood and TC-1 tumors were stained with phycoerythrin (PE)-conjugated HPV16 H-2D-RAHYNIVTF tetramer combined with surface staining using APC-Alexa Fluor-conjugated anti-CD8 (BD Pharmingen). Cells were analyzed on a BD FACSCalibur collecting 500,000 events.

### Intracellular cytokine staining and flow cytometry analysis

The cells harvested from the spleen of mice treated with various treatment regiments were incubated 0.1 μg/ml of HPV-16 E7 peptide containing an MHC class I epitope (aa49-57, RAHYNIVTF) in the presence of GolgiPlug (BD Pharmingen, San Diego, California, USA). The stimulated cells were then washed once with FACScan buffer and stained with phycoerythrin-conjugated monoclonal rat anti-mouse CD8a (clone 53.6.7). Cells were subjected to intracellular cytokine staining using the Cytofix/Cytoperm kit according to the manufacture’s instruction (BD Pharmingen). Intracellular IFN-γ was stained with FITC-conjugated rat anti-mouse IFN-γ. All antibodies were purchased from BD Pharmingen. Flow cytometry analysis was done using FACSCalibur with CellQuest software (BD Bioscience). FITC Rat IgG1, κ Isotype Control (Clone R3-34) was purchased from BD Pharmingen (Cat.# 554684).

### Statistical analysis

All data presented in this study are expressed as mean ± SD. At least three samples per group were included in each of these experiments. Flow cytometry data and results of tumor treatment experiments were evaluated by analysis of variance (1-way ANOVA) and the Tukey–Kramer test. Individual data points were compared by student’s *t*-test. For all analysis, *p* values <0.05 were considered significant.

### Additional file

**Additional file 1: Figure S1.** Characterization of antitumor effect in tumor bearing mice treated with intratumoral non-specific OVA long peptide vaccination in buccal mucosal region. 3 × 10^4^ TC-1-Luc cells were submucosally injected into the right buccal area of C57BL/6 mice (five per group). Three days after tumor injection, mice were vaccinated intratumorally with or without 50 μg of CTL peptide OVA241-270 for four times in a 4-day intervals. (A) Luminescence images of mice challenged with TC-1-Luc tumor and treated with or without OVA peptide vaccinations. (B) Line graph depicting the change in mean luminescence intensity of tumor bearing mice after tumor injection (mean ± SD). (C) Kaplan–Meier survival analysis of mice.

## Abbreviations

HPV: human papillomavirus
SLPs: synthetic long peptides
APCs: antigen presenting cells
CTL: cytotoxic T lymphocyte
I.T.: intratumoral
MHC: major histocompatibility complex
DCs: dendritic cells
IFN: interferon
