# Supplementary figures and images for: Buccal injection of synthetic HPV long peptide vaccine induces local and systemic antigen-specific CD8+ T-cell immune responses and antitumor effects without adjuvant
Source: Cell Biosci. 2016 Mar 3;6:17. doi: 10.1186/s13578-016-0083-9 (PMC4778350; doi:10.1186/s13578-016-0083-9)

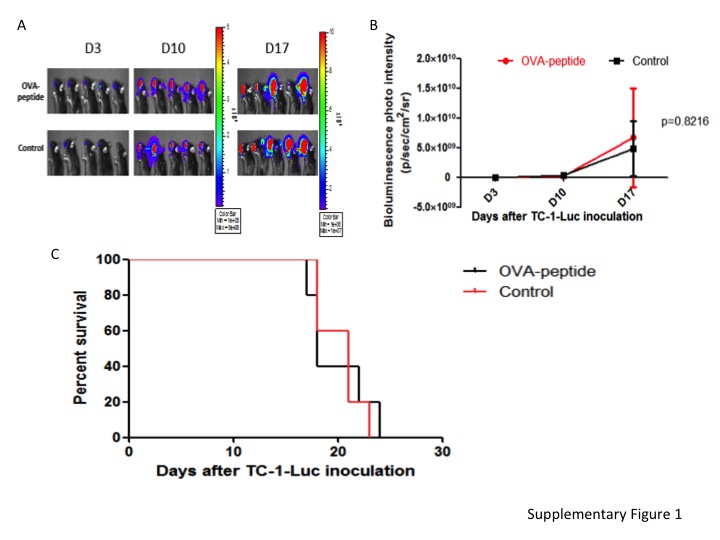

Supplement: Supplementary file 1 — Additional file 1: Figure S1. Characterization of antitumor effect in tumor bearing mice treated with intratumoral non-specific OVA long peptide vaccination in buccal mucosal region. 3 × 10^4^ TC-1-Luc cells were submucosally injected into the right buccal area of C57BL/6 mice (five per group). Three days after tumor injection, mice were vaccinated intratumorally with or without 50 μg of CTL peptide OVA241-270 for four times in a 4-day intervals. (A) Luminescence images of mice challenged with TC-1-Luc tumor and treated with or without OVA peptide vaccinations. (B) Line graph depicting the change in mean luminescence intensity of tumor bearing mice after tumor injection (mean ± SD). (C) Kaplan–Meier survival analysis of mice. [file 13578_2016_83_MOESM1_ESM.jpg]
